# Evaluating the impact of rubber mats in holding pens on fed cattle mobility and behavior at a commercial slaughter facility

**DOI:** 10.1093/jas/skaf288

**Published:** 2025-08-25

**Authors:** Lauren Dean, Huey Yi Loh, Paxton Sullivan, Carina Kautzky, Lacey Alexander, Lily Edwards-Callaway

**Affiliations:** Department of Animal Sciences, Colorado State University, Fort Collins, CO, 80524, USA; Department of Animal Sciences, Colorado State University, Fort Collins, CO, 80524, USA; Department of Animal Sciences, Colorado State University, Fort Collins, CO, 80524, USA; Department of Animal Sciences, Colorado State University, Fort Collins, CO, 80524, USA; Cargill, Wichita, KS, 67202, USA; Department of Animal Sciences, Colorado State University, Fort Collins, CO, 80524, USA

**Keywords:** cattle comfort, flooring, heifer, holding, steer, welfare, well-being

## Abstract

Animal welfare is an important consideration in livestock production, including the critical preslaughter period at processing facilities. While fed cattle spend a relatively short time in lairage, this phase presents a significant opportunity to mitigate stress and enhance well-being. The objective of this study was to determine the effects of providing rubber mats in holding pens on the mobility and behavior of fed cattle prior to slaughter. This study compared cattle held in pens with rubber mats (*n* = 92 pen groups) to those in control pens with stamped concrete flooring (*n* = 112 pen groups). Mobility was scored upon unloading and prior to stunning using a 4-point system (1 = normal to 4 = extremely reluctant to move). Pen-level behaviors (standing, lying, drinking, and motion) were quantified using instantaneous sampling at 5-min intervals for the duration of lairage. All instances of mounting were recorded. An employee survey was also administered to plant employees to gather their perspectives on the use of rubber mats in lairage (*n* = 13). Results indicated a tendency (*P* = 0.056) for cattle housed on rubber mats to have an increased odds of exhibiting normal mobility at the end of lairage compared to those on concrete. Cattle in matted pens exhibited greater odds of standing (OR: 1.5739, CI: 1.2807, 1.9351, *P* < 0.01) and mounting (OR: 1.9779, CI: 1.2194, 3.2072, *P* < 0.01), while showing lower odds of lying (OR: 0.5970, CI: 0.4727, 0.7531, *P* < 0.01) and drinking water (OR: 0.7500, CI: 0.6304, 0.8920, *P* < 0.01). Lairage duration and space allowance also influenced several behaviors. Notably, a large majority (92%) of plant employees preferred working in pens with rubber mats and believed they should be used in the holding pens to improve cattle movement during handling. These findings suggest that the incorporation of rubber mats in slaughter plant holding pens can potentially positively influence cattle mobility during lairage, potentially mitigating the decline in mobility often observed during the marketing process. The observed behavioral changes, including increased standing and mounting, may be indicative of improved comfort and stability on the softer, less slippery surface or increased stress; future work is needed to determine the motivation behind these behavioral changes. The strong positive feedback from plant employees further supports the potential benefits of this intervention for both animal welfare and worker well-being.

## Teaser

Installing rubber mats in holding pens in commercial slaughtering facilities could be a way to improve fed cattle comfort during the preslaughter period, as evidenced by a tendency to be associated with improved mobility post-lairage.

## Introduction

Animal welfare is an important animal management consideration in all livestock production systems. Although perceptions about livestock welfare differ globally (Prickett et al., 2010; Malek et al., 2017; Alonso et al., 2020), there is generally a shared expectation that production animals are humanely raised (which includes slaughter), which has subsequently influenced the way livestock are managed across supply chain sectors (Faucitano et al., 2022). Although fed cattle typically spend a relatively short amount of time in holding pens at slaughter facilities compared to dairy or feedlot settings, this period presents a critical opportunity to provide for their welfare. Reducing preslaughter stress through enhancing comfort in slaughter plant holding pens has the potential to positively influence animal welfare, meat quality, and production efficiency (Schwartzkopf-Genswein et al., 2012; Davis et al., 2022; Sullivan et al., 2022); many of these studies explore how changing certain preslaughter processes can impact animal welfare outcomes.

Quantifying cattle behavior is one way to assess animal welfare at the slaughter plant and has been integrated into many studies (reviewed by Davis et al., 2022). An animal’s ability to perform species-specific, highly motivated behaviors is a component of many animal welfare frameworks (the Five Freedoms, FAWC, 1993 ; Fraser’s Three Circles, Fraser et al., 1997; the Five Domains, Mellor et al., 2020). Among the quantifiable behaviors, mobility has been one of the most commonly used measurements in many studies to evaluate cattle welfare in a variety of production settings, including slaughter plants; studies evaluating cattle mobility upon unloading or during movement in the plant have reported a range of mobility impairment from 8% to 33% of cattle (Harris et al., 2018; Mijares et al., 2021; Borders et al., 2024, b, 2024; Sullivan et al., 2024). Not only is impaired mobility a welfare concern for cattle, but additionally, cattle in the slaughter plant with mobility issues are harder to move, thus increasing the risk of high-stress handling in the preslaughter period. Cattle that are hard to move can also potentially cause frustration in employees, causing dissatisfaction with their job and potentially leading to poor animal handling techniques, which could pose a human safety risk. In dairy and feedlot settings, the use of rubber matting in pens and drive alleys has been explored as a mechanism to improve cow comfort and often specifically as a mechanism to reduce lameness (Vanegas et al., 2006; Dewell et al., 2018; Sadharakia et al., 2019; Dawson et al., 2022). Although not yet formally explored, the use of rubber matting in holding pens at slaughter plants could be a way to enhance cattle comfort during lairage. We hypothesized that the incorporation of rubber mats at lairage would improve cattle mobility and alter behavior in the holding pens. This study aimed to investigate the effects of rubber mats in holding pens prior to slaughter on fed cattle mobility and behavior.

## Materials and Methods

### Ethical statement

The project procedures were observational and noninvasive, and the Colorado State University Institutional Animal Care and Use Committee (IACUC) issued a waiver (IACUC #4201) for this project. The survey component was reviewed and deemed exempt by the Institutional Research Board (IRB; # 4544).

### Facility

This study was conducted at a federally inspected slaughter facility in the Western region of the United States between May 2023 and March 2024. The facility operated 2 shifts, slaughtering approximately 4,000 cattle per day. Data were collected on 44 data collection days during the study period (12 spring days, 18 summer days, 9 fall days, and 5 winter days). Cattle arrived from 65 different feedyards and were all grain-finished. The average transport distance, estimated using the shortest route provided in Google Maps (Google, Mountain View, CA), was 266 ± 228 km with a minimum distance of 7 km and a maximum distance of 784 km. The majority of cattle were crossbred Angus, and there were some lots with Bos indicus influence. There were no Holsteins included in the study.

### Treatments

There were 2 treatments in this study: rubber mats in the holding pens and no rubber mats in the holding pens (control; stamped concrete). A total of 4 lairage pens were utilized for the study: 2 matted pens (259.76 and 259.19 m^2^) and 2 control pens (202.71 m^2^ and 222.97 m^2^). Pens with mats were selected due to their similar size and high-volume turnover during the shift to capture the maximum number of animals. Two different mat types (MaxGrip, thickness = 20.5 mm, length = 1.83 m, width = 1.22 m, diamond pattern texture; Chute mat, thickness = 28.58 mm, length = 1.8 m, width = 1.15 m, traction peg texture, Animat, Sherbrooke, QC, Canada) were used but the intent of the study was to evaluate the presence of mats as compared to no matting, therefore, mats were treated as one treatment for this experiment. The holding pens were outdoors with no cover, and during the summer months, sprinklers were used as heat stress mitigation for cattle as dictated by plant protocol. Sprinklers provided coverage to every pen, and all experimental pens experienced the same exposure throughout the course of the study. All flooring in the live animal handling areas of the facility was stamped concrete, except for the 2 experimental matted pens and the matted area on the unloading dock where the cattle exited the trailer.

Upon arrival at the slaughter facility, a group of cattle from one producer was allocated to an experimental pen based on plant scheduling logistics. Pen group (i.e., animals held in a holding pen together during lairage) was the experimental unit for the study. Each of the experimental pens could hold between 99 and 127 head of cattle. Upon arrival, origin of the cattle, average lot (i.e., producer group) weight, hide color (<25% of the pen was black-hided, 25% to 75% of the pen was black-hided, >75% of the pen was black hided), and sex class (i.e., heifer, steers, mixed steers and heifers) were collected for each pen group. The number of animals per pen group was recorded, and the space allowance per animal during lairage was calculated by dividing the total pen area by the number of animals per pen group; holding pens were not always filled to the maximum capacity. Lairage duration was estimated using the time the last group of animals entered the holding pen as the start time and the time that the first group of animals left the holding pen to be moved to the stunning area as the end time. Lairage time was determined by the plant schedule and was not experimentally controlled. Cattle were moved in smaller groups (approximately 40 animals) into the lairage pens, generally representing a truck load, and animals were moved out of the pen in smaller groups.

### Mobility scoring

Video cameras were used to capture footage of cattle upon movement to the holding pen as they exited the unloading pen (initial mobility score) and upon movement to the stunning area as they exited the holding pen at the end of lairage (final mobility score). A camera (HERO 5 and 10, GoPro, Inc., California, United States) was set up at each location to capture cattle’s initial and final mobility score. The video was analyzed by 2 researchers with extensive experience scoring cattle mobility at slaughter facilities; interobserver reliability was greater than 90% agreement between observers. Mobility was scored using the Meat Institute scoring system (1 = normal, walks easily, no apparent lameness, no change in gait; 2 = minor stiffness, shortness of stride, slight limp, keeps up with normal cattle; 3 = obvious stiffness, difficulty taking steps, obvious limp, obvious discomfort, lags behind normal cattle; 4 = extremely reluctant to move even when encouraged, statue-like; Meat Institute, 2015). The total number of cattle in each mobility category (i.e., 1 through 4) was summed, and the frequency of each score per pen group was calculated.

### Pen behavior

Video cameras (HERO 5 and 10, GoPro, Inc., California, USA) were set up to capture cattle behavior during lairage. Cameras were attached to extender poles (DocaPole, Tennessee, USA) and secured to existing fences approximately 2.44 m away from the entrance of each pen. The pole was extended approximately 7.62 m high to capture a complete view of the experimental pens. Video was analyzed by trained observers, who achieved >80% interobserver reliability, using instantaneous sampling with a 5-min scan interval (Bateson and Martin, 2021). At each observation interval, the number of cattle performing each behavior (standing, lying, and drinking water) was tallied and recorded as mutually exclusive events. For instance, cattle that were drinking were recorded as such, even if though they were also standing. The behaviors of interest are defined in Table 1. Behavior sampling was used to quantify mounting; one observer watched the video and recorded all instances of mounting that occurred within a pen group during the lairage period. A motion index was developed to quantify the number of animals moving during the holding period. Motion was defined as shifting weight between sides of the body and limbs or walking. At the 5-min scan intervals, motion was assessed using the following scale: 0: ≤ 1/3 of the cattle were moving; 1: > 1/3 of the cattle were moving.

**Table 1.** Pen behaviors of interest and their definition

Behavior	Definition
Lying^1^	The animal is recumbent, not supported by its legs
Standing	An animal is in an upright position supported by their legs, and can be walking or standing still.
Water drinking^1^	An animal has its head in or over the water trough.
Mounting	An animal positions the front half of its body on top of another animal’s topline using its front legs; attempted mounting was not included.
Motion	Weight shifting or walking; 0: < 1/3 of the cattle moving; 1: > 1/3 moving.

^1^Adapted from Meneses et al., 2021.

### Survey

Thirteen participants, including employees, supervisors, and superintendents, were asked to answer a survey to provide the extent of agreement with 4 statements (Table 2) regarding the usage of mats during lairage. The survey was administered 3 mo after the trial started. A researcher obtained each individual’s verbal consent prior to administering the survey. The survey was available in English or Spanish, and each participant could elect to take the written survey or have the survey read to them and provide their answers orally. Regarding each statement, participants could select from strongly agree, agree, disagree, or strongly disagree.

**Table 2.** Statements in the survey to evaluate the usage of mats during lairage

Question #	Statements
1	I prefer to work in holding pens with rubber mats compared to those without
2	Rubber mats should be used in holding pens.
3	Cattle seem to be more uncomfortable in holding pens with rubber mats compared to those without.
4	Cattle are more difficult to handle when coming out of the holding pens with rubber mats.

### Statistical analysis

All statistical analyses were performed in R statistical software version 4.3.2 and packages within R (R Core Team, 2023). A total of 22,949 cattle were included in the study; this included 112 experimental groups in the control (no mat) and 92 experimental groups in the matted treatment. One hundred and sixty-seven pens (18,833 total animals) and 159 pens (18,137 total animals) were used to assess the impact of rubber mats on mobility and behavior, respectively. The discrepancies in sample size between analyses were the result of the exclusion of experimental groups without complete data for all parameters (i.e., missing initial mobility score or missing space allowance information). For analysis, initial and final mobility scores were converted into a binary variable (0: normal or 1: abnormal); animals that scored 1 in mobility score were classified as normal, while animals that had a mobility score of 2 to 4 were classified as abnormal. The total number of animals that had normal and abnormal mobility scores was used as the response variable in the statistical analysis. Grouped binary logistic regression models were used for mobility and behaviors (lying, standing, and drinking); this model provides the probability of occurrence (i.e., normal mobility or displayed behavior), accounting for the total head per group. A generalized linear mixed model was fitted using Template Model Builder (Brooks et al., 2017) to evaluate the impact of rubber mat usage during lairage on the mobility of fed cattle. Initial mobility score (the number of animals with a normal initial mobility score in the pen group), total lairage time, and space allowance were included as covariates, and animals were included as the random effect in the model. The percentage of animals performing the measured behaviors was calculated for each lairage period by averaging the number of animals recorded as performing each behavior at each 5-min scan interval. Generalized linear models were fitted to investigate the impact of the utilization of a rubber mat on standing, lying, water drinking, and motion. Total lairage time and space allowance were included as covariates in the generalized linear models. The rate of mounting was calculated by dividing the total number of mounting events observed by the product of the total number of animals in the pen and the total duration time. A negative binomial generalized linear model was fitted to evaluate the effects of mat usage, sex, and their interaction on mounting behavior during lairage, with total lairage time and space allowance as covariates. After fitting the models for all variables, odds ratios were derived by exponentiating the estimated coefficients using the exponential function, which allows us to interpret the magnitude and direction of effects. The Likert (Bryer and Speerschneider, 2016) package was utilized to analyze and visualize the survey data, and a diverging bar chart was used to present the extent of agreement of plant employees on the usage of mats during lairage. Statistical significance was declared at *P* ≤ 0.05, while *P*-values between 0.05 and 0.10 were interpreted as a tendency.

## Results

Mixed sex groups (steers and heifers) were the most common sex class represented in the study population *(n* = 79, 38.73%), although by a relatively small margin (Table 3). The majority of experimental groups were >75% black-hided (*n* = 141, 69.12%). The average weight was 639.84 ± 41.77 kg.

**Table 3.** Sex class and hide color descriptions for the sample population (*n* = 204 pen groups)

Animal Characteristics (*n* = 204)	*n*	Frequency, %
**Sex**		
Heifers	56	27.45
Mixed	79	38.73
Steers	69	33.82
**Hide color**		
<25% black hided	17	8.33
25 to 75% black hided	46	22.55
>75% black hided	141	69.12

Table 4 includes the minimum, mean, and maximum lairage duration and space allowance for both the matted and unmatted pens. The mean lairage duration for the matted and unmatted pens was similar (272.90 ± 210.74 minutes and 322.58 ± 167.05 min, respectively). The mean space allowances were also similar between the matted and unmatted pens (2.08 ± 0.28 m^2^ and 2.14 ± 0.50 m^2^, respectively).

**Table 4.** The minimum, mean, and maximum time in lairage and space allowance during lairage for the sample population (*n* = 204)

Preslaughter Factors	Min	Mean	Max	SD
**No mats** (*n* = 112)				
Time in lairage (minutes)	54	322.58	804	210.74
Space allowance (m^2^/animal)	1.57	2.14	4.21	0.50
**With mats** (*n* = 92)				
Time in lairage (minutes)	45	272.90	812	167.05
Space allowance (m^2^/animal)	1.62	2.08	2.89	0.28

The frequencies of cattle exhibiting mobility scores of 1, 2, 3, or 4 before and after lairage are shown in Table 5. In both the matted and control groups, the majority of cattle (> 80%) had normal mobility (score of 1). The influence of mat usage, total lairage time, and space allowance on final mobility score is shown in Table 6. The utilization of rubber mats during lairage had a tendency (*P* = 0.056) to increase the odds of cattle having normal mobility when exiting the holding pen at the end of lairage. Lairage duration and space allowance did not have an impact (*P* > 0.10) on cattle mobility score.

**Table 5.** Frequency of fed cattle having mobility score 1 to 4 entering and exiting lairage pens with or without rubber mats

Variable	Frequency, %	
	No mats	With mats
*n* (animals)	9,435	9,398
Initial mobility score^1^		
1	82.01	82.67
2	17.58	16.93
3	0.39	0.40
4	0.00	0.00
Final mobility score^1^		
1	83.29	86.15
2	16.23	13.53
3	0.38	0.32
4	0.10	0.00

^1^Mobility was scored as either: 1 = normal, walks easily, no apparent lameness, no change in gait; 2 = minor stiffness, shortness of stride, slight limp, keeps up with normal cattle; 3 = obvious stiffness, difficulty taking steps, obvious limp, obvious discomfort, lags behind normal cattle; 4 = extremely reluctant to move even when encouraged, statue-like. Initial mobility score = mobility score upon unloading as they exited the unloading pen. Final mobility score = mobility score upon movement to the stunning area as they exited the holding pen at the end of lairage.

**Table 6.** Mobility score^1^ binary logistic regression analysis of fed cattle (*n* = 167 pen groups) housed in lairage pens with or without rubber mats

Variable	Estimate	SE^2^	Odds ratio^3^ (95% CI)	*P* value
Mat usage				0.056
No mats	*Referent*			
With mats	0.2792	0.1464	1.3221 (0.9907, 1.7654)	
Total lairage time, minutes	0.0001	0.0003	1.0001 (0.9907, 1.0007)	0.665
Space allowance, m^2^/animal	−0.1608	0.1954	0.8515 (0.5795, 1.2529)	0.411

^1^Mobility was originally scored as either: 1 = normal, walks easily, no apparent lameness, no change in gait; 2 = minor stiffness, shortness of stride, slight limp, keeps up with normal cattle; 3 = obvious stiffness, difficulty taking steps, obvious limp, obvious discomfort, lags behind normal cattle; 4 = extremely reluctant to move even when encouraged, statue-like. A mobility score of 1 was classified as normal, and a score of 2-4 was classified as abnormal for binary logistic regression analysis.

^2^SE, standard error.

^3^An odds ratio > 1 indicates that the variable is associated with a multiplicative increase in the odds of an animal showing normal mobility, whereas an odds ratio < 1 indicates that the variable is associated with a multiplicative decrease in the odds of an animal showing normal mobility.

The frequencies of fed cattle exhibiting standing, lying, drinking, moving, and mounting behaviors are shown in Table 7. The majority of cattle (>90%) were standing during lairage and more (*P* < 0.01) cattle were standing in rubber-matted lairage pens. Most pens (>65%) had less than 1/3 cattle moving in the lairage pen on average during the entirety of the lairage time. The mounting rate of cattle in the lairage pen with rubber mats was greater (*P* < 0.01) than cattle in the lairage pen without mats.

**Table 7.** Standing, lying, drinking, moving, and mounting behavior in lairage pens with or without rubber mats

Variable	No mats	With mats
n^1^ =	79	80
In-pen Behavior	Percentage of animals	
Standing	93.60	95.58
Lying	2.48	1.46
Drinking	3.40	2.54
	
Motion	Percentage of pen groups	
<1/3 are moving	65.82	70.00
>1/3 are moving	34.18	30.00
		
Mounting, rate per hour^2^	9.70	17.03

^1^The number of pens evaluated for cattle behavior. Cattle performing each behavior (standing, lying, and drinking water) were evaluated and recorded as mutually exclusive events. Motion was evaluated using the following scale: 0: ≤ 1/3 of the cattle were moving; 1: > 1/3 of the cattle were moving.

^2^All instances of mounting that occurred within a pen group during the lairage period were recorded, and the rate of mounting was calculated by $\frac{Total\;\;\;instances\;\;\;of\;\;\;mounting}{Total\;\;\;animal\;\;\;in\;\;\;the\;\;\;pen\;\;\;\times \;\;\;Lairage\;\;\;duration}\times 100$.

The influence of mat usage, total lairage time, and space allowance on cattle behavior is shown in Table 8. By using rubber mats in the holding pen, cattle had a greater odds of exhibiting standing (OR: 1.5739, CI: 1.2807, 1.9351, *P* < 0.01), while having lower odds of lying and drinking water (OR: 0.5970, CI: 0.4727, 0.7531, *P* < 0.01 and OR: 0.7500, CI: 0.6304, 0.8920, *P* < 0.01, respectively). Mat usage did not influence the odds (*P* > 0.10) of animals moving in the pens as determined by a motion index. A longer total lairage time was associated with fewer animals exhibiting standing (OR: 0.9932, CI: 0.9926, 0.9939, *P* < 0.05), drinking (OR: 0.9992, CI: 0.9983, 1.0000, *P* = 0.05), and moving (OR: 0.9959, CI: 0.9919, 0.9995, *P* < 0.01), while increasing the chance of animals lying (OR: 1.0074, CI: 1.0068, 1.0081, *P* < 0.01). A greater space allowance led to less animals standing and moving (OR: 0.5274, CI: 0.4447, 0.6284, *P* < 0.01, and OR: 0.1131, CI: 0.0264, 0.3863, *P* < 0.01, respectively) in the pen and increased the odds of lying (OR: 1.8392, CI: 1.5181, 2.2148, *P* < 0.01) in the holding pen. Space allowance did not impact (*P* > 0.10) the odds of animals exhibiting water drinking behavior.

**Table 8.** Logistic regression of behavior^1^ performed by fed cattle in holding pens (*n *= 159) with or without rubber mats during lairage

Variable	Estimate	SE^2^	Odds ratio (95% CI)^3^	*P* value
*Standing*				<0.01
Mat usage				
No mats	*Referent*			
With mats	0.4536	0.1052	1.5739 (1.2807, 1.9351)	
Total lairage time, minutes	−0.0068	0.0003	0.9932 (0.9926, 0.9939)	<0.01
Space allowance, m^2^/animal	−0.6398	0.0882	0.5274 (0.4447, 0.6284)	<0.01
*Lying*				
Mat usage				<0.01
No mats	*Referent*			
With mats	−0.5159	0.1187	0.5970 (0.4727, 0.7531)	
Total lairage time, minutes	0.0074	0.0003	1.0074 (1.0068, 1.0081)	<0.01
Space allowance, m^2^/animal	0.6093	0.0962	1.8392 (1.5181, 2.2148)	<0.01
*Water*				
Mat usage				
No mats	*Referent*			
With mats	−0.2877	0.0885	0.7500 (0.6304, 0.8920)	0.01
Total lairage time, minutes	−0.0008	0.0004	0.9992 (0.9983, 1.0000)	0.05
Space allowance, m^2^/animal	−0.0418	0.1209	0.9591 (0.7511, 1.2070)	0.728
*Motion*				
Mat usage				
No mats	*Referent*			
With mats	−0.1603	0.3621	0.8519 (0.4181, 1.7381)	0.658
Total lairage time, minutes	−0.0041	0.0019	0.9959 (0.9919, 0.9995)	0.026
Space allowance, m^2^/animal	−2.1793	0.6861	0.1131 (0.0264, 0.3863)	<0.01

^1^Standing, lying, and drinking water were recorded as mutually exclusive events. Motion was defined as shifting weight between sides of the body and limbs or walking, and motion was assessed using the following binary scale: 0: ≤ 1/3 of the cattle were moving; 1: > 1/3 of the cattle were moving.

^2^SE, standard error.

^3^An odds ratio > 1 indicates that the variable is associated with a multiplicative increase in the odds of an animal performing a certain behavior, whereas an odds ratio < 1 indicates that the variable is associated with a multiplicative decrease in the odds of an animal performing a certain behavior.

The influence of mat usage, sex, their interaction, total lairage time, and space allowance on mounting behavior is shown in Table 9. There were main effects of mat usage (*P* = 0.023) and sex (*P* = 0.016) on mounting behavior during lairage, with a tendency (*P* = 0.068) for interaction between the two factors. When rubber mats were used during lairage, holding pens with only heifers (OR: 4.159, CI: 1.453, 11.904, *P* = 0.004) and pens with only steers (OR: 6.37, CI: 2.232, 18.182, *P* < 0.01) had a greater mounting rate compared to holding pens with both steers and heifers (mixed). When mats were not applied during lairage, there were no differences (*P* > 0.10) in mounting rate between sex groups. Holding pens with only steers showed a lesser mounting rate (OR: 0.359, CI: 0.176, 0.733, *P* = 0.005) when mats were not used compared to when mats were used during lairage. The mounting rate in the mixed and heifers pens did not differ (*P* > 0.10) with the usage of mats. Cattle had a greater mounting rate (OR: 1.004, CI: 1.001, 1.006, *P* = 0.002) when they spent more time in the holding pens, and greater space allowance tended (OR: 1.641, CI: 1.029, 2.780, *P* = 0.069) to have a greater mounting rate during lairage.

**Table 9.** Logistic regression of mounting behavior^1^ performed by fed cattle in holding pens (*n *= 159) with or without rubber mats during lairage

Variable	Estimate	SE^2^	Odds ratio (95% CI)^3^	P value
Mats*sex				0.068
Mat usage				0.023
No mats				
Heifer vs Mixed	0.3962	0.751	1.486 (0.455 to 4.856)	0.713
Heifer vs Steers	0.0178	0.399	1.018 (0.406 to 2.552)	0.999
Steers vs Mixed	0.3784	0.316	1.46 (0.495 to 4.310)	0.691
With mats				
Heifer vs Mixed	1.4252	1.87	4.159 (1.453 to 11.904)	0.004
Heifer vs Steers	0.4267	0.256	0.653 (0.261 to 1.634)	0.521
Mixed vs Steers	1.8519	0.07	6.369 (2.232 to 18.182)	<0.01
Sex				0.016
Heifer				
No mats vs with mats	−0.581	0.234	0.559 (0.246 to 1.272)	0.166
Mixed				
No mats vs with mats	0.448	0.819	1.566 (0.562 to 4.365)	0.392
Steers				
No mats vs with mats	−1.025	0.131	0.359 (0.176 to 0.733)	0.005
Time spent in pen, minutes	0.004	0.001	1.004 (1.001 to 1.006)	0.002
Space allowance, m^2^/animal	0.495	0.272	1.641 (1.029 to 2.780)	0.069

^1^Animal positions the front half of its body on top of another animal’s topline using its front legs; attempted mounting was not included.

^2^SE, standard error.

^3^An odds ratio > 1 indicates that the variable is associated with a multiplicative increase in the odds of an animal performing a certain behavior, whereas an odds ratio < 1 indicates that the variable is associated with a multiplicative decrease in the odds of an animal performing a certain behavior.

**Figure 1.**
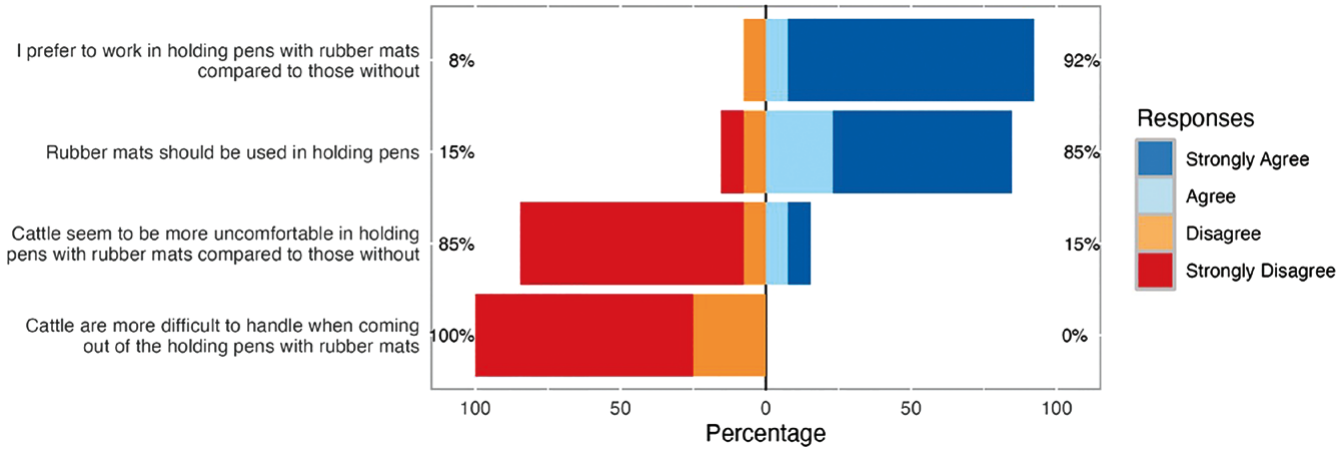
The extent of agreement on each survey statement among workers (*n* = 13) in the slaughter plant regarding the usage of rubber mats during lairage.

Figure 1 summarizes the survey results from 13 plant employees on their extent of agreement regarding the statements related to mat usage during lairage. Ninety-two percent of participants (*n *= 12) agreed or strongly agreed that they “prefer to work in holding pens with rubber mats compared to those without”, and 85% of participants (*n* = 11) agreed or strongly agreed “that rubber mats should be used in the holding pens”. Eighty-five percent of participants (*n* = 11) disagreed or strongly disagreed that “cattle seem to be more uncomfortable in holding pens with rubber mats compared to those without”, and all the participants disagreed or strongly disagreed that “cattle are more difficult to handle when coming out of holding pens with rubber mats”.

## Discussion

In the United States, ensuring cattle welfare at slaughter is a critical component of plant management systems and is generally governed by federal regulations, internal company policies, and customer requirements. Companies practice low-stress handling, provide training to animal handlers, and try to create an environment to minimize stress during the preslaughter period (personal communication, L. Edwards-Callaway). Over the past decade, the focus on cattle mobility at the slaughter plant has grown (Harris et al., 2018; Mijares et al., 2021; Borders et al., 2024, b, 2024; Sullivan et al., 2024). Cattle mobility is considered a key welfare indicator, but it can also impact plant productivity, i.e., if cattle have poor mobility, they are more difficult to move and slow down the efficiency of the slaughtering process. Despite cattle mobility being a welfare priority at slaughter, to date, we have not identified any studies evaluating how the alteration of the pen surface using rubber mats during lairage could improve cattle mobility before slaughter. In the United States, cattle generally do not remain in lairage at the plant for an extended period of time; lairage duration has been reported to be approximately 3 hours in finished cattle (Eastwood et al., 2017; Davis et al., 2024b) and 6 hours in culled cows and bulls (Borders et al., 2024). Lairage duration can vary depending on several factors, including but not limited to truck arrival times (e.g., do trucks arrive late or early) and plant efficiencies (e.g., chain speeds). In lairage, there is often limited space to lie down and animals may be fatigued from transport. Rubber mats in holding pens at slaughter facilities may provide a softer surface to reduce pressure and improve comfort prior to slaughter. In the current study, the use of rubber matting in lairage pens as a mechanism to improve cattle comfort prior to slaughter was evaluated. Rubber mats had a tendency to improve the odds of cattle having normal mobility at the end of lairage, identifying that the addition of rubber mats into the holding pens at slaughter plants may be a way to improve mobility impairment and increase cattle comfort. This finding should be interpreted with caution as it was a tendency, but it does support future work in this area.

In the current study, the majority (>80%) of cattle had normal mobility in both treatments (mats and no mats) and also at both time points (initial and final); this frequency is within the range reported in several other studies that have quantified cattle mobility at commercial slaughter plants without mats (Harris et al., 2018; Lee et al., 2018; Mijares et al., 2021; Borders et al., 2024, b, 2024; Sullivan et al., 2024). Mobility at slaughter can be impacted by many different factors, including animal characteristics (e.g., weight and sex class; Mijares et al., 2021), environmental characteristics (e.g., temperature-humidity index, windspeed, season; Mijares et al., 2021; Davis et al., 2024a; Sullivan et al., 2024), and preslaughter management procedures (e.g., distance hauled, truck waiting time, space allowance during lairage; Mijares et al., 2021; Davis et al., 2024a; Sullivan et al., 2024). Additionally, several studies without mats in lairage pens have demonstrated an increase in mobility impairment as cattle progress through the marketing process (i.e., from the feedyard, upon unloading at the plant, and after lairage; Boyd et al., 2015; Hagenmaier et al., 2017a, b).

The use of rubber mats in the holding pens at the facility in this study had a tendency to increase the odds of cattle having normal mobility at the end of the lairage period. As noted, other studies have indicated an increase in compromised mobility over time in the marketing process (Boyd et al., 2015; Hagenmaier et al., 2017a, b), and there is great potential for rubber mats to both ameliorate and combat impaired mobility during lairage. Additionally, space allowance and time in lairage, 2 factors that may impact mobility, did not have an association with mobility score in this study. Research on dairies (Vanegas et al., 2006; Chapinal et al., 2013; Bergsten et al., 2015) and indoor feedyards (Dewell et al., 2018; Dawson et al., 2022) has also demonstrated an improvement in mobility in cattle that are housed on rubber mats compared to ones that are not. Dawson et al. (2022) demonstrated that indoor feedlot cattle raised on concrete slatted flooring had worse mobility, evaluated using the Zinpro Step-Up Locomotion Scoring System (Zinpro, 2023), as compared to cattle housed in the same environment with various rubber mat types, one type being the kind that was utilized in the current study. Mobility score did not differ by utilizing different mat types used in the current study (data not shown), but that could be included in future research to identify the optimal mat for the application. Similarly, Dewell et al. (2018) reported increased locomotion score and lameness frequency in cattle housed on slatted concrete floors as compared to the flooring with rubber mats. Studies have also measured heel erosion, claw disorders, and leg lesions, demonstrating a decrease in prevalence with rubber matting (Vanegas et al., 2006; Bergsten et al., 2015); these parameters were not measured in the current study as cattle were only in the plant for a short period of time but depending on the application, including these types of animal-based outcomes in future work could be valuable. Interestingly, in the current study, there were no cattle with a mobility score of 4 upon arrival at the plant, but at the end of lairage, there were 0.01% (9 out of 9,435), notably a very low percentage, and they were all in the control group (unmatted pens). Perhaps exploring the impacts of rubber mats on high-risk animals would be another area to explore.

Despite lairage being a relatively short time period, it is important to provide animals with the opportunity to express highly motivated behaviors. Although many studies have measured behavior as a welfare indicator at slaughter (Davis et al., 2022), the past research has focused more on behavior events or actions, like vocalization, falling, and agonistic interactions, rather than characterizing how cattle spend their time during lairage, and thus, this study could provide a benchmark for future work. In the case of slaughter plants, important highly motivated behaviors could be drinking, rumination, lying down to help with rest and recovery from transport. In this study, lying, standing, drinking, and motion of the pen group were quantified, and the presence of rubber mats in the holding pens was associated with the change in percentages of animals standing, lying, and drinking, but not in motion. In both the matted and unmatted pens, the majority of cattle (>90%) were standing during the lairage period. Cattle had higher odds of standing and lower odds of lying and drinking water in the matted pens. It is unclear why cattle in the matted pens were more likely to be standing; although only speculative, perhaps the softer rubber surface in the pens provided more comfort to cattle, and standing was more tolerable and less uncomfortable. It should be noted that even though more cattle were standing in this study, lying is a highly motivated behavior in cattle (Tucker et al., 2018), so likely there are other factors impacting behavioral expression in lairage. Cattle may have preferred to lie, but did not have the opportunity to do so due to limited space. The average space allowances between matted and unmatted pens were similar, so pen density alone likely does not explain the difference noted. As previously discussed, the cattle’s mobility also improved over lairage, so rubber mats could have provided some relief to their hooves and legs during the lairage period, which influenced their behavior. The sample population in this study included some groups of cattle that were held in the lairage pens overnight (40 of the 204 pen groups were held overnight), but it would be beneficial to do targeted observation on these cattle, as they generally have more space and are held in lairage for longer periods of time.

The rate of mounting was greater in the matted pens but was influenced by sex class. It should be noted that mounting is presented as a rate, but was generally a behavior performed by only a few animals, i.e., based on anecdotal observations, most cattle did not mount during lairage, and some cattle accounted for the majority of mounting. Mounting can be an expression of sexual behavior and also agonistic behavior. To the best of the authors’ knowledge, there are no current studies reporting mounting behavior in slaughter environments, but previous work in dairy cattle on-farm has shown that flooring type (e.g., dirt vs concrete vs rubber mats) influences the frequency of mounting behavior (Vailes and Britt, 1990; Platz et al., 2008). Platz et al. (2008) evaluated estrus behavior in dairy cows housed on rubber-matted flooring and found that cows on the matted flooring mounted more and slipped less during mounting as compared to those on the concrete slatted floors with no mats. Vailes and Britt (1990) reported an interaction between floor type and the presence of estrual cows. Excessive mounting can be a welfare problem. The buller steer syndrome, i.e., one or more animals in a pen are repeatedly mounted by several cattle causing deleterious effects on the animals being ridden, often observed in feedyards is thought to be impacted by many factors including but not limited to social hierarchy, seasonal effects, weather, floor space, group size, and stress (reviewed by Blackshaw et al., 1997). Stressors such as a change in routine and an increase in pen activity could impact the expression of bullying behavior in groups of cattle. Increased mounting is often observed when unfamiliar animals are mixed and animals work to establish and maintain a stable social hierarchy (Tennessen et al., 1985; Kenny and Tarrant, 1987; Raj et al., 1991). In the case of this study, cattle were not mixed and remained with contemporary groups from the feedyard, but perhaps the stress of the new environment impacted the expression of mounting behavior. Some researchers suggest that mounting behavior is impacted by stocking density (i.e., the greater the proximity between animals, the greater the opportunity for mounting; Tennessen et al., 1985), and in this study, there was no association between space allowance and mounting behavior. Excessive mounting during lairage in cattle could pose a concern for animal welfare (i.e., injury, additional stress) and should be monitored but in the context of this study objective, the authors speculate that the expression of this behavior may have been related to improved stability and sure footing, i.e., the rubber mats were less slippery, which is why it was seen more in the matted pens.

In the United States, federal regulations require that livestock have access to water in the holding pens ( CFR, 1979 ). However, the regulations do not have any requirements for space allowance except during overnight holding, when animals must have adequate space to lie down (CFR, 1979). There are industry guidelines for space allowance in the holding pens (The Meat Institute, 2021) that slaughter plants often incorporate into best management practices. The mean space allowance in lairage for finished cattle in the United States that was reported in a large benchmarking study was 3.1 ± 2.0 m^2^ (Davis et al., 2024b), and thus, the behavioral diversity expressed by cattle during lairage is often limited due to space. Space allowance and lairage duration were associated with both lying and standing behavior. As space allowance and lairage duration increased, albeit in a small magnitude of change, the odds of cattle standing decreased, and the odds of cattle lying increased. Lying down requires more area, and thus it is logical that as cattle had more space, more cattle were able to engage in lying behavior. As lairage duration increased, perhaps cattle became more fatigued and prioritized resting by lying down. Resting behavior is a highly motivated behavior in cattle, particularly when cattle have been deprived of the ability to do so (Tucker et al., 2018), which would be the case in cattle that have just been transported to a slaughtering facility. Future work could also consider different lying positions (e.g., sternal, lateral) to evaluate if differences exist. Interestingly, the number of animals drinking was not influenced by space allowance but was influenced by the lairage duration. It should be noted that in this study, “drinking” included animals that had their heads over the waterer in addition to those actually drinking. There were 2 waterers in each holding pen at this facility, so the cattle had to navigate through the pen to gain access.

Movement was evaluated using a motion index developed by the authors. It was noted during study planning that while many cattle did not necessarily walk due to limited space, they did spend time in motion, shifting weight side to side or taking a few steps in either direction. Both space allowance and lairage duration impacted cattle motion. As space allowance increased, the odds of more cattle being in motion decreased; a similar relationship was seen with lairage duration, but to a substantially smaller degree. Practical methods of evaluating cattle movement or latency to settle down should be explored to utilize in research in slaughter plant settings.

The results of the survey that was included as part of this study indicated that the majority of the employees both preferred working on the mats and felt that mats should be used in the holding pens. Although this was not quantified formally, anecdotally, employees shared that they felt the cattle could lie down and get up more easily on the mats in addition to moving more calmly because the mats were not as slippery. Slipping was not measured in this study, but Cozzi et al. (2013) reported that finishing bulls raised on rubber mats slipped less than bulls on fully slatted concrete floors. None of the employees felt cattle were more difficult to move on the matted pens (as compared to unmatted); they also indicated the mats provided them (the employees) with better footing and comfort while working. The authors recognize that this is a small sample size of individuals, but the sample population is significant, representing the individuals who work at this particular facility and interact with thousands of cattle daily. The addition of mats into holding pens at a slaughter plant would represent a potentially significant change for the individuals working there. Across industries, implementing change is a challenging task requiring a strategic approach to implement successful change (Kotter, 2012). If employees do not feel they have organizational support (i.e., their organization values their contribution and well-being), they can experience reduced commitment and increased absenteeism (Eisenberger et al., 1986); if employees identify perceived organizational support, it will enhance the affiliation they have for the organization, organizational commitment, and their well-being ( Eisenberger et al., 2020). Engaging employees in discussions regarding potential changes can be instrumental to understanding their sentiments and reactions to changes, which both demonstrates a commitment to employee well-being and also assists in addressing any concerns proactively (Hubbart, 2024). Additionally, by promoting the well-being of both the cattle and the employees in this environment, we are cultivating a more sustainable beef production system. When feasible, it would be valuable to gather feedback from animal caretakers when investigating how new procedures or technologies may impact animal welfare.

The addition of rubber mats into the holding pens at slaughter plants could be a way to improve cattle welfare during the preslaughter period, which is a particular benefit of improving mobility. Additionally, perspectives shared by individuals at the facility were positive, with employees indicating support for the use of mats due to both employee and animal benefits. There are logistical considerations that plants should consider when implementing mats, such as cleaning procedures, costs, and repair. Economic investment will vary by mat type and number of mats needed, so plants should evaluate the cost:benefit ratio to determine the optimal plan for their facility; maintenance costs should also be considered when evaluating long-term costs. In this study, 2 different mats were used, but as no differences were identified during preliminary statistical exploration, the 2 mat types were combined; future work should explore differences between mat types, as there may be different outcomes related to mat thickness and groove pattern. Some slaughter plants have started exploring the value of having mats in the lairage pens, but the adoption is still recent, and therefore, the durability of mats will need to be considered in that particular environment. Although this work sampled a representative population across seasons (i.e., weather), times of day associated, producers, and distances traveled, future work should consider a deeper exploration of some of these factors.

## Abbreviations

IACUC: Institutional Animal Care and Use Committee
IRB: Institutional Review Board

## References

[CIT0001] Alonso, M. E., J. R. González-Montaña, and J. M. Lomillos. 2020. Consumers’ concerns and perceptions of farm animal welfare. Animals. 10:385. doi: https://doi.org/10.3390/ani10030385 32120935 PMC7143148

[CIT0002] Bateson, M., and P. R. Martin. 2021. Measuring behaviour: an introductory guide. 4th ed. Cambridge University Press, Cambridge, United Kingdom.

[CIT0003] Bergsten, C., E. Telezhenko, and M. Ventorp. 2015. Influence of soft or hard floors before and after first calving on dairy heifer locomotion, claw and leg health. Animals. 5:662–686. doi: https://doi.org/10.3390/ani5030378 26479380 PMC4598700

[CIT0048] Blackshaw, J. K., A. W. Blackshaw, and J. J. McGlone. 1997. Buller steer syndrome review. Appl. Anim. Behav. Sci. 54:97–108.

[CIT0004] Borders, S. E., T. E. Schwartz, T. R. Mayer, K. B. Gehring, D. B. Griffin, C. R. Kerth, K. E. Belk, L. Edwards-Callaway, J. A. Scanga, M. N. Nair, et al. 2024. National Beef Quality Audit—2022: transportation, mobility, live cattle, and hide assessments to determine producer-related defects that affect animal welfare and the value of market cows and bulls at processing facilities. Transl Anim Sci 8:txae033. doi: https://doi.org/10.1093/tas/txae033 38616995 PMC11015891

[CIT0005] Boyd, B. M., S. D. Shackelford, K. E. Hales, T. M. Brown-Brandl, M. L. Bremer, M. L. Spangler, T. L. Wheeler, D. A. King, and G. E. Erickson. 2015. Effects of shade and feeding zilpaterol hydrochloride to finishing steers on performance, carcass quality, heat stress, mobility, and body temperature1. J. Anim. Sci. 93:5801–5811. doi: https://doi.org/10.2527/jas.2015-9613 26641190

[CIT0006] Brooks, M. E., K. Kristensen, K. J. van Benthem, A. Magnusson, C. W. Berg, A. Nielsen, H. J. Skaug, M. Mächler, and B. M. Bolker. 2017. glmmTMB balances speed and flexibility among packages for zero-inflated generalized linear mixed modeling. R J 9:378–400. doi: https://doi.org/10.32614/RJ-2017-066

[CIT0046] Bryer, J., and K. Speerschneider. 2016. likert: Analysis and Visualization Likert Items. R package version 1.3.5. https://CRAN.R-project.org/package=likert

[CIT0050] Code of Federal Regulations, Title 9, pt. 313, 1979. eCFR :: 9 CFR Part 313 -- Humane Slaughter of Livestock. Available at: https://www.ecfr.gov/current/title-9/chapter-III/subchapter-A/part-313 (Accessed August 27, 2025).

[CIT0009] Chapinal, N., A. K. Barrientos, M. A. G. Von Keyserlingk, E. Galo, and D. M. Weary. 2013. Herd-level risk factors for lameness in freestall farms in the northeastern United States and California. J. Dairy Sci. 96:318–328. doi: https://doi.org/10.3168/jds.2012-5940 23141819

[CIT0010] Cozzi, G., E. Tessitore, B. Contiero, R. Ricci, F. Gottardo, and M. Brscic. 2013. Alternative solutions to the concrete fully-slatted floor for the housing of finishing beef cattle: Effects on growth performance, health of the locomotor system and behaviour. Vet. J. 197:211–215. doi: https://doi.org/10.1016/j.tvjl.2013.03.001 23607913

[CIT0011] Davis, M., P. Sullivan, J. Bretón, L. Dean, and L. Edwards-Callaway. 2022. Investigating the impact of pre-slaughter management factors on indicators of fed beef cattle welfare – a scoping review. Front. Anim. Sci. 3:1073849. doi: https://doi.org/10.3389/fanim.2022.1073849

[CIT0012] Davis, M. K., P. A. Sullivan, A. M. Hess, M. N. Nair, D. F. Mooney, and L. N. Edwards-Callaway. 2024a. An analysis of the influence of preslaughter management factors on welfare and meat quality outcomes in fed beef cattle in the United States. Transl Anim Sci 8:txae108. doi: https://doi.org/10.1093/tas/txae108 39119361 PMC11306929

[CIT0013] Davis, M. K., P. A. Sullivan, A. M. Hess, M. N. Nair, D. F. Mooney, and L. N. Edwards-Callaway. 2024b. Benchmarking current preslaughter management factors, welfare indicators, and meat quality outcomes at commercial fed cattle processing facilities in the United States. Transl Anim Sci 8:txad150. doi: https://doi.org/10.1093/tas/txad150 38259258 PMC10803158

[CIT0014] Dawson, C. R., P. A. Henley, A. R. Schroeder, W. T. Meteer, C. A. Hayes, T. L. Felix, D. W. Shike, and J. C. McCann. 2022. Effects of rubber matting on feedlot cattle growth performance, locomotion, and carcass characteristics in slatted floor facilities. J. Anim. Sci. 100:skac041. doi: https://doi.org/10.1093/jas/skac041 35148402 PMC9030117

[CIT0015] Dewell, R. D., G. A. Dewell, R. M. Euken, L. J. Sadler, C. Wang, and B. A. Carmichael. 2018. Association of floor type with health, well-being, and performance parameters of beef cattle fed in indoor confinement facilities during the finishing phase. Bov. Pract. 52:16–25. doi: https://doi.org/10.21423/bovine-vol52no1p16-25

[CIT0016] Eastwood, L. C., C. A. Boykin, M. K. Harris, A. N. Arnold, D. S. Hale, C. R. Kerth, D. B. Griffin, J. W. Savell, K. E. Belk, D. R. Woerner, et al. 2017. National Beef Quality Audit-2016: transportation, mobility, and harvest-floor assessments of targeted characteristics that affect quality and value of cattle, carcasses, and by-products. Transl Anim Sci 1:229–238. doi: https://doi.org/10.2527/tas2017.0029 32704647 PMC7250433

[CIT0017] Eisenberger, R., R. Huntington, S. Hutchison, and D. Sowa. 1986. Perceived organizational support. J. Appl. Psychol. 71:500–507. doi: https://doi.org/10.1037//0021-9010.71.3.500

[CIT0051] Eisenberger, R., Rhoades Shanock, L. and Wen, X., 2020. Perceived organizational support: why caring about employees counts. Annu. Rev. Organ. Psychol. Organ. Behav. 7:101–124.

[CIT0019] Faucitano, L., G. Martelli, E. Nannoni, and X. Manteca. 2022. Fundamentals of animal welfare in meat animals and consumer attitudes to animal welfare. p. 667–703 in New aspects of meat quality. Elsevier, Amsterdam, The Netherlands.

[CIT0052] FAWC. Farm Animal Welfare Council. 1993. Report on priorities for research and development in farm animal welfare. Available at: https://edepot.wur.nl/134980. (Accessed May 16, 2025).

[CIT0020] Fraser, D., D. M. Weary, E. A. Pajor, and B. N. Milligan. 1997. A scientific conception of animal welfare that reflects ethical concerns. Anim. Welf. 6:187–205. doi: https://doi.org/10.1017/s0962728600019795

[CIT0021] Hagenmaier, J. A., C. D. Reinhardt, S. J. Bartle, J. N. Henningson, M. J. Ritter, M. S. Calvo-Lorenzo, G. J. Vogel, C. A. Guthrie, M. G. Siemens, and D. U. Thomson. 2017a. Effect of handling intensity at the time of transport for slaughter on physiological response and carcass characteristics in beef cattle fed ractopamine hydrochloride12. J. Anim. Sci. 95:1963–1976. doi: https://doi.org/10.2527/jas.2016.0821 28727025

[CIT0022] Hagenmaier, J. A., C. D. Reinhardt, M. J. Ritter, M. S. Calvo-Lorenzo, G. J. Vogel, C. A. Guthrie, M. G. Siemens, K. F. Lechtenberg, D. J. Rezac, and D. U. Thomson. 2017b. Effects of ractopamine hydrochloride on growth performance, carcass characteristics, and physiological response to different handling techniques1,2. J. Anim. Sci. 95:1977–1992. doi: https://doi.org/10.2527/jas.2016.0936 28726982

[CIT0023] Harris, M. K., L. C. Eastwood, C. A. Boykin, A. N. Arnold, K. B. Gehring, D. S. Hale, C. R. Kerth, D. B. Griffin, J. W. Savell, K. E. Belk, et al. 2018. National Beef Quality Audit–2016: assessment of cattle hide characteristics, offal condemnations, and carcass traits to determine the quality status of the market cow and bull beef industry. Transl Anim Sci 2:37–49. doi: https://doi.org/10.1093/tas/txx002 32704688 PMC7200876

[CIT0024] Hubbart, J. A. 2024. Organizational change: implications of directive change management. Hum. Res. Manage. Serv. 6:3457. doi: https://doi.org/10.18282/hrms.v6i2.3457

[CIT0025] Kenny, F. J., and P. V. Tarrant. 1987. The behaviour of young Friesian bulls during social re-grouping at an abattoir. Influence of an overhead electrified wire grid. Appl. Anim. Behav. Sci. 18:233–246. doi: https://doi.org/10.1016/0168-1591(87)90219-x

[CIT0026] Kotter, J. P. 2012. Leading Change. [Nachdruck], with a new preface by the author. Harvard Business Review Press, Boston.

[CIT0027] Lee, T. L., C. D. Reinhardt, S. J. Bartle, E. F. Schwandt, M. S. Calvo-Lorenzo, C. Vahl, J. A. Hagenmaier, M. J. Ritter, G. J. Vogel, and D. U. Thomson. 2018. An epidemiological investigation to determine the prevalence and clinical manifestations of slow-moving finished cattle presented to slaughter facilities. Transl. Anim. Sci. 2:241–253. doi: https://doi.org/10.1093/tas/txy056 32704708 PMC7200404

[CIT0044] Malek, L., W. J. Umberger, and J. Rolfe. 2017. Segmentation of Australian meat consumers on the basis of attitudes regarding farm animal welfare and the environmental impact of meat production. Anim. Prod. Sci. 58:424. doi:10.1071/an17058

[CIT0030] Mellor, D. J., N. J. Beausoleil, K. E. Littlewood, A. N. McLean, P. D. McGreevy, B. Jones, and C. Wilkins. 2020. The 2020 five domains model: including human–animal interactions in assessments of animal welfare. Animals. 10:1870. doi: https://doi.org/10.3390/ani10101870 33066335 PMC7602120

[CIT0045] Meneses, X. C. A., R. M. Park, E. E. Ridge, and C. L. Daigle. 2021. Hourly activity patterns and behaviour-based management of feedlot steers with and without a cattle brush. Appl. Anim. Behav. Sci. 236:105241. doi:10.1016/j.applanim.2021.105241

[CIT0029] Mijares, S., M. Calvo-Lorenzo, N. Betts, L. Alexander, and L. N. Edwards-Callaway. 2021. Characterization of fed cattle mobility during the COVID-19 pandemic. Animals. 11:1749. doi: https://doi.org/10.3390/ani11061749 34208118 PMC8230808

[CIT0031] Platz, S., F. Ahrens, J. Bendel, H. H. D. Meyer, and M. H. Erhard. 2008. What happens with cow behavior when replacing concrete slatted floor by rubber coating: a case study. J. Dairy Sci. 91:999–1004. doi: https://doi.org/10.3168/jds.2007-0584 18292255

[CIT0032] Prickett, R., F. B. Norwood, and J. Lusk. 2010. Consumer preferences for farm animal welfare: results from a telephone survey of US households. Anim. Welf. 19:335–347. doi: https://doi.org/10.1017/s0962728600001731

[CIT0033] R Core Team. 2023. R: A language and environment for statistical computing. R Foundation for Statistical Computing, Vienna, Austria. Available from: https://www.R-project.org/ (Accessed May 15, 2025).

[CIT0034] Raj, A. M., B. W. Moss, W. J. McCaughey, W. McLauchlan, D. J. Kilpatrick, and S. J. McGaughey. 1991. Behavioural response to mixing of entire bulls, vasectomised bulls and steers. Appl. Anim. Behav. Sci. 31:157–168. doi: https://doi.org/10.1016/0168-1591(91)90002-F

[CIT0035] Sadharakiya, K., L. Sorathiya, A. Raval, G. Sabapara, and P. Patel. 2019. Effects of rubber mat flooring on hygiene, locomotion, hock and knee injury in crossbred cows. Int. J. Livest. Res. 1:1. doi: https://doi.org/10.5455/ijlr.20181026050531

[CIT0036] Schwartzkopf-Genswein, K. S., L. Faucitano, S. Dadgar, P. Shand, L. A. González, and T. G. Crowe. 2012. Road transport of cattle, swine and poultry in North America and its impact on animal welfare, carcass and meat quality: a review. Meat Sci. 92:227–243. doi: https://doi.org/10.1016/j.meatsci.2012.04.010 22608833

[CIT0038] Sullivan, P., M. Davis, J. Bretón, and L. Edwards-Callaway. 2022. Investigating the impact of pre-slaughter management factors on meat quality outcomes in cattle raised for beef: a scoping review. Front. Anim. Sci. 3:1065002. doi: https://doi.org/10.3389/fanim.2022.1065002

[CIT0037] Sullivan, P. A., M. K. Davis, M. N. Nair, A. M. Hess, D. F. Mooney, and L. N. Edwards-Callaway. 2024. Preslaughter factors affecting mobility, blood parameters, bruising, and muscle pH of finished beef cattle in the United States. Transl Anim Sci 8:txae035. doi: https://doi.org/10.1093/tas/txae035 38562213 PMC10983080

[CIT0049] Tennessen, T., M. A. Price, and R. T. Berg. 1985. The social interactions of young bulls and steers after re-grouping. Appl. Anim. Behav. Sci. 14:37–47.

[CIT0039] The Meat Institute. 2015. Mobility Scoring for Cattle. Available from: https://www.youtube.com/watch?v=QIslfHCvkpg (Accessed May 15, 2025).

[CIT0040] The Meat Institute. 2021. Recommended Animal Handling Guidelines & Audit Guide: A Systematic Approach to Animal Welfare. Available from: https://www.meatinstitute.org/sites/default/files/original%20documents/Animal_Handling_Guide_English.pdf (Accessed May 16, 2025).

[CIT0041] Tucker, C. B., L. Munksgaard, E. M. Mintline, and M. B. Jensen. 2018. Use of a pneumatic push gate to measure dairy cattle motivation to lie down in a deep-bedded area. Appl. Anim. Behav. Sci. 201:15–24. doi: https://doi.org/10.1016/j.applanim.2017.12.018

[CIT0042] Vailes, L. D., and J. H. Britt. 1990. Influence of footing surface on mounting and other sexual behaviors of estrual Holstein cows. J. Anim. Sci. 68:2333–2339. doi: https://doi.org/10.2527/1990.6882333x 2401655

[CIT0043] Vanegas, J., M. Overton, S. L. Berry, and W. M. Sischo. 2006. Effect of rubber flooring on claw health in lactating dairy cows housed in free-stall barns. J. Dairy Sci. 89:4251–4258. doi: https://doi.org/10.3168/jds.S0022-0302(06)72471-7 17033012

[CIT0047] Zinpro. 2023. Locomotion Scoring of Beef Cattle. [Accessed May 15, 2025]. https://www.zinpro.com/wp-content/uploads/2023/04/Zinpro_Locomotion_Scoring_BeefCattle.pdf

